# Geographic Distribution and ORF5 Diversity of PRRSV-2 Variants in Midwestern U.S. Diagnostic Submissions, 2023–2025

**DOI:** 10.3390/pathogens15070710

**Published:** 2026-07-07

**Authors:** Ramchander Nadipelly, Mohamed Selim, Jagathiswaran Radhakrishnan, Gun Temeeyasen, Eric Nelson, Travis J. Clement, Naveen Duhan, Sunil K. Mor

**Affiliations:** 1 Department of Veterinary and Biomedical Sciences, South Dakota State University, Brookings, SD 57007, USA; ramchander.nadipelly@sdstate.edu (R.N.); mohamed.selim@sdstate.edu (M.S.); jagathiswaran.radhakrishnan@sdstate.edu (J.R.); gun.temeeyasen@sdstate.edu (G.T.); eric.nelson@sdstate.edu (E.N.); 2 Animal Disease Research and Diagnostic Laboratory, South Dakota State University, Brookings, SD 57007, USA; travis.clement@sdstate.edu

**Keywords:** ORF5 region, Sanger sequencing, PRRSV, lineage, variants, epitopes

## Abstract

Porcine reproductive and respiratory syndrome (PRRS) is a serious disease that causes reproductive failure and late abortion in breeder farms, as well as severe respiratory manifestations in pigs of all ages. In the United States, the swine industry has been affected by PRRSV-2, particularly due to control challenges and the virus’s ongoing evolution. In this study, we conducted a genetic characterization of the 642 PRRSV-2 ORF5 sequences generated from diagnostic submissions at the Animal Disease Research and Diagnostic Laboratory (ADRDL) at South Dakota State University (SDSU). These sequences were generated over a 3-year period from 2023 through 2025, with 208 (32.4%), 130 (20.2%), and 304 (47.4%) sequences in 2023, 2024, and 2025, respectively. These sequences were from swine diagnostic submissions from ten states in the Midwest, including Minnesota (MN; 350/642, 54.5%), South Dakota (SD; 182/642, 28.3%), and Iowa (IA; 73/642, 11.4%), collectively accounting for 94.2% of submitted sequences. The genetic typing revealed that the L1C sub-lineage was the most frequently detected among the submitted sequences, accounting for around 60%, with L1C.5 as the major contributor (371/642). Other sub-lineages, such as L5A, L1A, L1D, and L1H, were also detected, but in smaller numbers. More than 80% of the detected sequences were classified as wild-type or wild-like based on ORF5 identity to commercial vaccine reference strains. Interestingly, this study documented increased detection of the recently reported L1C.5.32 variant, which was the most frequently detected variant in 2025 (123 detections), particularly in Minnesota. Moreover, L1C.5.34, L1C.5.36, L1C.3.25, and L1C.2 variants were also detected, and the L1H.18 variant showed increased detection, especially in 2025. In conclusion, this study provides genetic characterization of PRRSV-2 ORF5 sequences from Midwestern U.S. diagnostic submissions (a key U.S. swine production region), submitted to the SDSU ADRDL.

## 1. Introduction

The Porcine reproductive and respiratory syndrome (PRRS) disease has a devastating economic impact on the global pig industry. PRRS remains one of the most economically important diseases affecting the U.S. swine industry, with recent estimates suggesting approximately $1.2 billion in annual losses, based on data from 2016 to 2020 [1]. PRRSV, a member of the family *Arteriviridae*, genus *Porartevirus*, is an enveloped, positive-sense, single-stranded RNA virus with an approximately 15 kb-long genome [2]. The PRRSV genome comprises 10 open reading frames (ORFs), including ORF1a, 1b, 2a, 2b, 3, 4, 5a, 5, 6, and 7. ORF1a and ORF1b encode two large polyproteins that generate 14 nonstructural proteins [3]. The eight remaining ORFs (ORF2a, ORF2b, ORF3–7, and ORF5a) are responsible for the generation of structural proteins, including glycoprotein (GP) 2, small envelope (E), GP3, GP4, GP5, membrane (M), nucleocapsid (N), and ORF5a proteins, respectively [4].

Two distinct genotypes of PRRSV were isolated around the same time in both North America (VR2332) and Europe (Lelystad virus) in the early 1990s [5]. Despite their high pathogenicity similarity, the two PRRSV-2 types showed only 65% nucleotide identity. Thus, they were later classified into two separate species: *Betaarterivirus suid* 1 (PRRSV-1) and *Betaarterivirus suid* 2 (PRRSV-2) [6]. Currently, genotyping and classification of PRRSVs are primarily based on nucleotide sequences of ORF5 and ORF7 [7]. ORF5 encodes GP5, a transmembrane envelope protein composed of 603 nucleotides, which is highly divergent across various PRRSV strains and has a high mutation rate (~0.5–1% per year). It can be used efficiently for genotyping [7,8].

GP5 is the major viral protein that harbors neutralizing epitopes, which plays a crucial role in inducing virus-neutralizing antibodies. Specific residues in the ORF5-encoded GP5 protein are the primary targets for neutralizing antibodies [9,10,11,12]. Mutations in these regions have been associated with altered immune recognition. PRRSV-2 GP5 contains both decoy and neutralizing epitopes at the positions comprising residues 27–30 (^27^VLVN^30^) and residues 37–45 (^37^SHLQLIYNL^45^) [9]. Additionally, two hypervariable regions, HVR-1 (32–35) and HVR-2 (56–60), were reported previously [13]. Two previously recognized B cell epitopes (aa 1–15 and aa 187–200) and two T cell epitopes (aa 117–131 and aa 149–163) are related to the host immune response against PRRSV-2 [14,15]. Positive selection pressure analysis confirmed 18 codon sites in GP5. Four positive selection sites at positions 2, 5, 6, and 15 were involved in the B cell epitope region (positions 1–15), two positive selection sites at residues 28 and 30 were included in the decoy epitope region (positions 27–30), and five positive selection sites at residues 32, 33, 34, 35, and 59 were incorporated in HVR-1 and 2. Moreover, two additional positive selection sites (102 and 104) located in the transmembrane region and sites 39,137,151,153 and 196 are located in the primary neutralizing epitope (positions 37–45) [9], T-cell epitopes (positions 149–163), and the C-terminal region, respectively [13]. Changes in these antigenic regions of GP5 may contribute to viral diversification and potential antigenic differences.

Furthermore, the PRRSV GP5 ectodomain possesses variable N-glycosylation (N-gly) sites, which have been associated with glycan shielding, immune recognition, and infectivity [16,17]. Eight potential N-glycosylation sites have been recognized in PRRSV-2: N30, N32, N33, N34, N35, N44, N51, and N57. Interestingly, the positions and numbers of these glycosylation sites exhibit several distinct patterns (A-F). Patterns A (33-44-51) and B (34-44-51) are the most abundant patterns that are widely distributed in the USA [18]. Changes in one or more glycosylation sites result in significant modifications to the conformational structure of epitopes, allowing the virus to circumvent host immunity [19].

Based on ORF5 nucleotide sequences, two primary methods have been widely used for PRRSV-2 classification: restriction fragment length polymorphism (RFLP) analysis and sequencing [20]. RFLP analysis primarily used three or more restriction enzymes to digest the PCR-amplified ORF5 sequence. Based on the digestion pattern of each enzyme, an RFLP code was assigned to the sequence [21]. It was used effectively to predict the genotypic relationship among various field strains and to differentiate between vaccine and field strains [21,22,23]. However, some challenges affected its reliability, such as closely related viruses having different RFLP patterns [24] or other divergent viruses may share the same RFLP pattern (e.g., RFLP 1-8-4, 1-4-2) [17]. Hence, the efficacy of RFLP analysis for molecular characterization of field PRRSVs is limited.

An alternative classification of PRRSV-2 is based on the phylogenetic relationships of ORF5 sequences. Firstly, a 2010 classification grouped PRRSV-2 into 9 lineages, with the potential for further emergence of new lineages and sub-lineages. The genetic divergence among the various lineages is approximately 17% [20]. Recently, a new lineage and sub-lineage classification of PRRSV-2 was proposed [25] which is more comprehensive, fine-scaled, and flexible. According to this proposed classification, PRRSV-2 was classified into 11 lineages (L1-L11), with further subdivision into 21 sub-lineages. Secondly, phylogenetic analysis and nucleotide identity between ORF5 sequences and those of available commercial vaccines are also helpful for classifying PRRSV-2 into vaccine-like and wild strains [26].

Vaccination is the primary means of controlling PRRSV infection in pig farms. Several commercial live attenuated and inactivated vaccines have been used extensively in swine farms at all ages to reduce the severity of infection and virus shedding compared to unvaccinated pigs [27,28]. So far, six commercially modified live virus (MLV) vaccines, which are attenuated live viruses, have been approved for use on swine farms in the USA [25]. However, the use of live attenuated vaccines has led to the coexistence of both vaccinal and field strains within the same farm and host, increasing the possibility of recombination, a normal process in RNA viruses. This condition led to the emergence of recombinant PRRSV-2 variants with the unique ability to circumvent immunity conferred by most commercial vaccines [21].

In this study, we performed genetic analysis of ORF5 sequences obtained from clinical samples submitted to the Animal Disease Research and Diagnostic Laboratory (ADRDL) at South Dakota State University (SDSU) from swine farms in the Midwest reporting PRRSV cases over the past three years. The main purpose of this study was to investigate the genetic diversity of PRRSV detected among Midwestern diagnostic submissions from 2023 through 2025 by conducting in-depth molecular characterization of ORF5 sequences.

## 2. Materials and Methods

### 2.1. Clinical Samples

A total of 642 PRRSV-2 ORF5 sequences were retrieved from the database of Animal Disease Research and Diagnostic Laboratory (ADRDL) at South Dakota State University (SDSU) from 2023 through 2025. These sequences were generated from clinical samples tested as part of routine diagnostic submissions at ADRDL and were confirmed positive for PRRSV-2 by realtime RT-PCR before being selected for ORF5 sequencing and downstream genetic analysis. The total number of sequences included in this study was 208 from 2023, 130 from 2024, and 304 from 2025. These sequences represented PRRSV-2 infection in swine farms across multiple U.S. states, primarily from Minnesota (*n* = 350), South Dakota (*n* = 182), and Iowa (*n* = 73) with additional submissions from Colorado (*n* = 12), Illinois (*n* = 11), Nebraska (*n* = 7), Wisconsin (*n* = 4), and Kansas, Tennessee, and Virginia (*n* = 1 each). The cycle threshold (Ct) values for these RT-PCR-positive samples ranged from 18 to 30. Because this was a diagnostic-submission dataset, sequence counts were interpreted as detections among submitted samples rather than population-based incidence estimates.

### 2.2. Sequencing

The PRRSV-2 ORF5 sequences were generated by Sanger sequencing [29]. Sequencing was performed at the SDSU-ADRDL using the BigDye Terminator v3.1 Cycle Sequencing Kit (Applied Biosystems, Vilnius, Lithuania), followed by purification with the BigDye Xterminator Purification Kit (Applied Biosystems, Bedford, MA, USA), according to the manufacturer’s instructions. The recovered sequences ranged from 603 to 606 nucleotides, corresponding to the full length of ORF5.

### 2.3. Phylogenetic Analysis

Phylogenetic analysis was performed for ORF5 sequences obtained in the present study (*n* = 642; 2023–2025) and 32 PRRSV ORF5 reference lineage strains [25] and 6 vaccine strain sequences collected from GenBank. Sequences were aligned using MAFFT [30], and phylogenetic trees were reconstructed using the Maximum-Likelihood (ML) method implemented in IQ-TREE version 3 [31]. The best-fitting nucleotide substitution model was determined using ModelFinder [32], with GTR+I+R5 selected as the optimal model according to the Bayesian Information Criterion. Branch support was assessed using the ultrafast bootstrap approximation with 1000 replicates, together with SH-aLRT branch tests (1000 replicates). Phylogenetic trees were visualized and annotated using iTOL [33].

Lineages and sublineages were assigned by using the PRRSV-Loom variants web tool (https://stemma.shinyapps.io/PRRSLoom-variants/) (accessed on 2 May 2026) [34], which applies a machine-learning classifier trained on curated ORF5 datasets to generate standardized variant identifiers. These lineage assignments were further cross-validated against the phylogenetic topology to confirm the lineage designations. Because classification frameworks and reference datasets continue to evolve, variant assignments were interpreted within the limits of the ORF5-based classification system. Variant frequencies were summarized by year and state to describe patterns of detection and variant turnover among submitted PRRSV-2 sequences. Vaccine relatedness was assessed by comparing field ORF5 sequences with vaccine reference strains using the refined ORF5 identity-based classification described by Yim-Im et al. (2023) [25], categorizing sequences as vaccine-like (≥98% identity), vaccine-like suspect (95 < 98%), or wild-type (<95%). This analysis was conducted by incorporating the 642 ORF5 sequences from this study along with reference sequences from commercially available PRRSV-2 modified-live vaccines, including Ingelvac PRRS MLV (2-5-2; sub-lineage L5A), Ingelvac PRRS ATP (1-4-2; sub-lineage L8A), Fostera PRRS (1-3-2; L8C), Prime Pac PRRS RR (1-4-4; L7), Prevacent PRRS (1-8-4; L1D), and PRRSGard (1-8-4; L1F). To improve epidemiological resolution beyond basic lineage and sublineage classifications, each ORF5 sequence was also assigned to a fine-scale variant using VanderWaal et al.’s [34] dynamic nomenclature. This system segments PRRSV-2 diversity into genetically consistent, surveillance-oriented units that can adapt to emerging variants. The translated ORF5 amino acid sequences (*n* = 642) were aligned using MAFFT, and possible N-glycosylation sites in GP5 were identified using a custom Python 3.12.13 script. Each GP5 sequence was 200 amino acids in length and screened for the consensus N-X-S/T motif, where X represented any amino acid except proline, and the presence or absence of detected sites was used to define glycotype patterns across the dataset. Formal recombination inference was not performed because the study was based on ORF5 sequences. Due to the limited genomic region analyzed, ORF5 data were not used to infer definitive recombination breakpoints, parental strains, or genome-wide mosaic patterns. For exploratory comparison of submitted-sequence composition, contingency tables were used to compare major-lineage and top-variant distributions across years. Expected cell counts were examined, and because some expected counts were <5, Fisher–Freeman–Halton exact tests for r × c tables were performed using Monte Carlo simulation with 100,000 replicates. Cramer’s V was calculated to describe the strength of association.

## 3. Results

### 3.1. Classification of the Newly Detected ORF5 into Lineages, Sub-Lineages, and Groups

**Figure 1:**
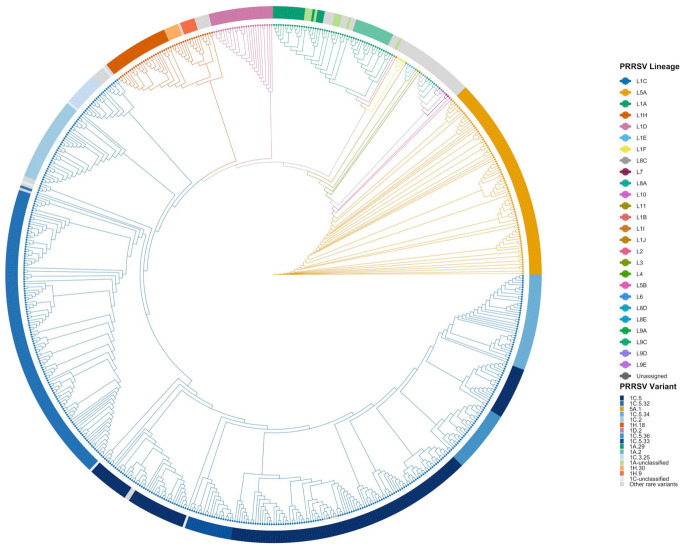
Circular maximum-likelihood phylogenetic tree constructed from 642 PRRSV ORF5 nucleotide sequences. Branch colors indicate PRRSV lineages, while the outer ring denotes variant or sub-lineage classifications. Displayed bootstrap values indicate supported nodes, while weakly supported internal branches were not emphasized. The radial layout illustrates the genetic diversity and clustering of PRRSV isolates across major lineages and variants.

A total of 642 PRRSV-2 ORF5 sequences related to diagnostic submissions from 2023 to 2025 were classified by comparison with representative reference sequences spanning established PRRSV-2 lineages and sub-lineages. Classification showed a structured ORF5 dataset, mainly consisting of Lineage 1 (L1), especially the L1C sub-lineage, which accounted for over 60% of the submitted ORF5 sequences. The most common was L1C.5 with 371 out of 642 samples, followed by L1C.2 and L1C.3 with 35 and 17 out of 642, respectively. Within the available dataset, additional L1 sub-lineages were observed at relatively low frequencies, including L1A, L1H, L1D, L1F, and L1E, comprising 53, 45, 25, 3, and 3 sequences, respectively. The other sub-lineages identified among the detected sequences were L5A (*n* = 82). By contrast, lineages L8C and L7 were represented by only 2 and 1 sequences, respectively (Figure 1). To support the interpretation of the phylogenetic clustering, the number of sequences assigned to each lineage/variant is summarized in Supplementary Figure S1. Refined identification of each detected sub-lineage and group using the PRRSLoom-variants web tool revealed further classification into distinct variants or subgroups. This classification was clear in L1C.5, which included classical L1C.5 (*n* = 162) plus a distinct emergence of subsequent variants, including L1C.5.32 (*n* = 123), L1C.5.34 (*n* = 39), L1C.5.36 (*n* = 25), and L1C.5.33 (*n* = 19). For L1C.3 sub-lineage, the L1C.3.25 variant was the predominant (*n* = 14), while for L1C.2 sub-lineage, the L1C.2 variant (*n* = 33) was detected. The emergence of variants was also evident in L1H, with L1H.18 (*n* = 27) the most common, followed by L1H.30 (*n* = 6). L1A displayed two main variants: L1A.29 (*n* = 17) and L1A.2 (*n* = 16). However, in ORF5 aligned with L5A, the L5A.1 variant was the only detected variant, comprising all detected strains (*n* = 82). The classification of the detected sequences was supported by ORF5 phylogenetic clustering and PRRSLoom assignment. Selected lineage- or variant-associated clades showed moderate to high bootstrap support, whereas deeper relationships among lineages were interpreted cautiously.

In addition to the tree topology, the identity matrix for the created sub-lineages, groups, and variants was also consistent and supported the topology of the reconstructed ML tree. The identity percentage was calculated between the aligned sequences and the reference ORF5, and between the aligned sequences themselves. The identity percentages for the aligned ORF5 sequences of the L1C.5, L1C.3, and L1C.2 groups ranged from 91.87 to 100% nucleotides (nt) and 90.5% to 100% amino acids (aa), 91.21% to 99.83% nt and 91% to 100% aa, and 83.08% to 100% nt and 82.5% to 100% aa, respectively. The identity percentages of the new ORF5 sequences that clustered with reference sequences from other L1 sub-lineages were as follows: 90.55% to 100% nt and 89.5% to 100% aa with L1A, 97.01–100% nt and 95% to 100% aa with L1D; 85.74% to 99.83% nt and 88% to 100% aa with L1H; 88.89–98.51% nt and 89–97% aa with L1E; 97.84–99.83% nt and 96–100% aa with L1F. The identity percentage between the aligned sequences with the reference L5A sequence was 96.68–100% nt and 94–100% aa, and it was 99.34% nt and 98% aa between the aligned sequence with the reference L8C ORF5 sequence (Table 1). Regarding the divergence analysis of the generated sub-lineages and groups, low average distances (4.25% for L1C.5; 5.5% for L1C.3; 3.66% for L1C.2) were observed. Meanwhile, the average distance within the created sub-lineages also varied, from very low (0.81) in the L5A sub-lineage to high (8.61) in the L1H sub-lineage, which exhibited the highest divergence.

### 3.2. Distribution of PRRSV-2 Sub-Lineages in Midwest Diagnostic Submissions (n = 642)

The genetic analysis of the sequences shows a clear predominance of Lineage 1 (L1), which constitutes 86.7% (557/642) of the dataset. Lineage 5 (L5), particularly L5A, is the second most common lineage, accounting for 82 of 642 sequences (12.8%). According to the dataset, minor lineages such as L8C and L7 were represented at very low frequencies, comprising 2 and 1 sequences, respectively. Within L1, sub-lineage L1C, particularly the L1C.5 group, is the main contributor to genetic diversity, accounting for 371 sequences (57.7%), followed by sub-lineages L1A (8.25%; 53/642) and L1H (7.01%; 45/642). The most frequently detected variants were L1C.5 (25.2%; 162/642), L1C.5.32 (19.2%; 123/642), and L5A.1 (12.8%; 82/642), respectively. These combined represent 57.2% of all detections. Secondary contributors were L1C.5.34 (6.1%; 39/642), L1C.2 (5.1%; 33/642), and L1H.18 (4.2%; 27/642), along with many low-frequency lineages like L1E, L1F, L7, and L8 (each ≤ 1%). These detections exhibited geographic concentration within the submitted diagnostic dataset, and should not be interpreted as population-level evidence of nationwide circulation. In Minnesota, the majority of detected ORF5 sequences were clustered with variants of L1C.5.32 (30%; 105/350), L1C.5 (20.86%; 73/350), L5A.1 (11.71%; 41/350), L1H.18 (6.29%; 22/350), L1D.2 (4.29%; 15/350), L1C.5.34 (3.71%; 13/350), and L1A.29 (2.86%; 10/350). In South Dakota, most of the identified ORF5 sequences were aligned with the following strains: L1C.5 (31.87%; 58/182), L1C.5.34 (12.09%; 22/182), L5A.1 (11.54%; 21/182), L1C.2 (7.69%; 14/182), and L1C.5.32 (7.14%; 13/182). In Iowa, the ORF5 strains detected were assigned as L1C.5 (41.1%; 30/73), L1C.2 (13.7%; 10/73), and L5A.1 (8.22%; 6/73). Within submitted samples, this indicated that the most frequently detected variants of the L1C sub-lineage in Minnesota were L1C.5.32, followed by L1C.5, while in South Dakota, it was L1C.5, followed by L1C.5.34.

**Figure 2:**
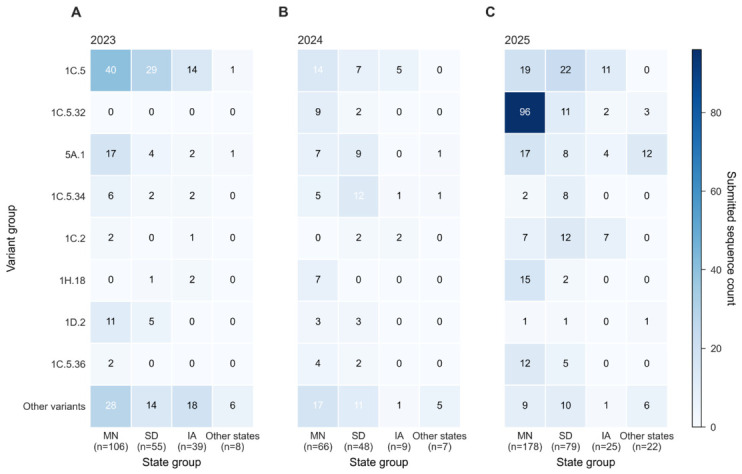
Distribution of major PRRSV-2 ORF5 variant groups by year and state group among diagnostic submissions. Panels show submitted sequence counts for (**A**) 2023, (**B**) 2024, and (**C**) 2025. State-group totals are shown below each column. Counts are descriptive diagnostic-submission summaries and should not be interpreted as population-level incidence.

The yearly distribution of PRRSV ORF5 sequences was mainly dominated by lineage L1C. In 2023 (*n* = 208), L1C was the most common lineage at 55.77% (116/208), followed by L1A at 17.99% (37/208), L5A at 11.54% (24/208), L1D at 7.69% (16/208), and L1H at 5.77% (12/208). In 2024 (*n* = 130), the share of L1C increased to 63.85% (83/130), while L5A was at 13.08% (17/130), L1H at 10.77% (14/130), L1A at 5.38% (7/130), and L1D at 4.62% (6/130). This trend continued in 2025 (*n* = 304), with L1C representing 75.33% (229/304) of all sequences. In 2025, L5A was 13.49% (41/304), L1H was 6.25% (19/304), L1A was 2.96% (9/304), and L1D was 0.99% (3/304). Overall, the distribution of PRRSV ORF5 sequences over the years was led by lineage L1C, which comprised 116 sequences in 2023, 83 in 2024, and 229 in 2025, totaling 428 across all three years. The second most common lineage was L5A, with 24 (2023), 17 (2024), and 41 (2025) sequences, totaling 82. Lineage L1A ranked third, with 37 (2023), 7 (2024), and 9 (2025), for a total of 53 sequences. Lineage L1H included 12 (2023), 14 (2024), 19 (2025), and 45 sequences overall. The least represented lineage was L1D, with 16 (2023), 6 (2024), and 3 (2025) sequences, totaling 25. Although other sub-lineages, such as L1E, L1F, L8C, and L7, had fewer sequences, they still contributed to overall diversity over the years. The temporal distribution of detected groups and variants changed from 2023 to 2025. Although the L1C.5 group was predominant among diagnostic submissions throughout the three years of the study, its variants or subgroups exhibited distinct temporal patterns. For example, L1C.5 showed a decrease in detections from its 2023 peak (*n* = 84) to 26 in 2024, then increased slightly to 52 in 2025. On the other hand, L1C.5.32 increased among submitted sequences, from no detection in 2023 to a slight increase in 2024 (*n* = 11) and a larger number of detections in 2025 (*n* = 112), becoming the most frequently detected variant that year. Whereas the L1C.5.36 variant occurred mainly in 2025 (*n* = 17). Similarly, lineage L5A.1 grew over time (2023: 24 detections, 2024: 17, 2025: 41). Figure 2 summarizes the distribution of major variant groups by year and state group among submitted sequences. The distribution of submitted ORF5 sequences differed by year when grouped by major lineage (Fisher-Freeman-Halton test with Monte Carlo simulation, *p* < 0.001; Cramer’s V = 0.231). This result was interpreted as an exploratory comparison of submitted-sequence composition rather than as evidence of population-level incidence, because the dataset lacked population denominators and repeat-sampling information, as presented in the Supplementary File S1.

### 3.3. Amino Acid Analysis of ORF5

The multiple sequence alignment of 371 ORF5 sequences from the L1C.5 group showed extensive nonsynonymous mutations in HVR1 (32–35) and HVR2 (56–60). In addition to these common mutations in the L1C.5 group, the created subgroups or variants contained specific distinguishing mutations. For example, L1C.5.36 exhibited characteristic substitutions in the B cell epitope (10–15 aa), T cell epitope (at residue K163R), decoy epitope at the positions comprising residues 27–30, and the V77I mutation. Moreover, L1C.5.33 showed two significant mutations, F23S and T98I, compared with the other variants.

The L1C.3-aligned ORF5 sequences revealed multiple nonsynonymous mutations in the B epitope (1–15), the decoy epitope (27–30), and the neutralizing epitopes (37–45) compared to the reference sequence. Additionally, characteristic substitutions in HVR-1 (32–35) and HVR-2 (56–60), as well as at positive selection sites 104 and 200 amino acids in the B epitope, were observed. In the L1C.2 aligned ORF5 sequences, nonsynonymous mutations were observed at residues 34 in HVR-1 and 58 in HVR-2. The 53 ORF5 sequences aligned with the L1A sub-lineage showed notable nonsynonymous mutations in aa 3 and 15 of the B epitopes (1–15), HVR-1, HVR-2, positive pressure selection sites 102 and 104, residue 158 in the T epitope, and residues 191 and 192 in the B epitope, compared to the L1A reference ORF5 sequence.

The 25 ORF5 sequences aligned with the L1D sub-lineage exhibited few nonsynonymous amino acid substitutions in HVR-1, with no other mutations in the antigenic regions of ORF5. Regarding the 45 ORF5 sequences aligned with the L1H sub-lineage, multiple nonsynonymous substitutions were observed at residues 3, 11, and 15, as well as 191 and 192 in the B epitope sites and both decoy and neutralizing epitopes at the positions comprising residues 27–30 and residues 37–45. Moreover, substitutions were observed in the T cell epitope region (positions 149–163) and residues 57, 58, and 59 in HVR-2. The variants created in L1H showed characteristic mutations; L1H.18 variant strains carried two additional distinct mutations, V72A and L87F, compared to the other variants.

### 3.4. N-Linked Glycosylation Sites

**Figure 3:**
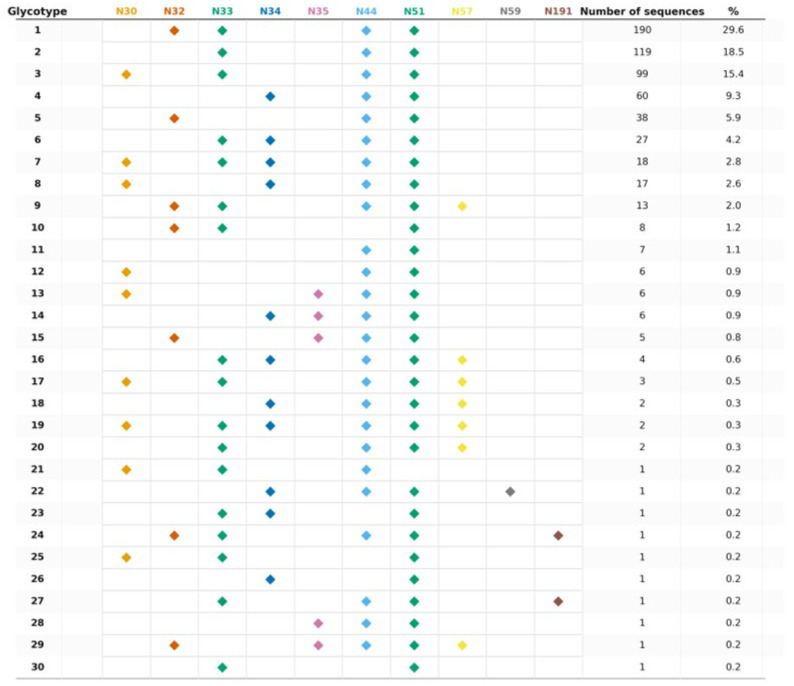
Distribution of glycotypes among analyzed sequences. Each row represents a distinct glycotype defined by the presence of predicted N-linked glycosylation sites at positions N30, N32, N33, N34, N35, N44, N51, N57, N59, and N191. Colored diamonds indicate the presence of a glycosylation motif. The number and percentage of sequences corresponding to each glycotype are shown on the right. Glycotype 1 was the predominant pattern (190 sequences, 29.6%), followed by glycotypes 2 (18.5%) and 3 (15.4%). Conserved glycosylation was mainly observed at N44 and N51, whereas variability occurred primarily at positions N30–N35 and N57–N191.

N-glycosylation analysis identified 10 predicted N-glycosylation sites in the 642 ORF sequences detected in this study, including N30, N32, N33, N34, N35, N44, N51, N57, N59, and N191. Based on these sites, the sequences were grouped into 30 distinct glycotypes. The distribution showed variable patterns, with a few dominant glycotypes comprising most sequences. Glycotype 1, defined by N32, N33, N44, and N51, was the most common, accounting for 190 sequences (29.6%). This was followed by glycotypes 2 (N33, N44, and N51; 18.5%), 3 (N30, N33, N44, and N51; 15.4%), 4 (N34, N44, and N51; 9.3%) and 5 (N32, N44 and N51; 5.9%). Collectively, the top five glycotypes made up nearly 79% of all sequences, indicating that most strains share a limited number of key glycosylation patterns. Sites N44 and N51 were highly conserved across nearly all glycotypes, suggesting they form the main glycosylation backbone, while variation at N30, N32, N33, and N34 contributed significantly to glycotype differences. Conversely, N35, N57, N59, and N151 were found only in minor glycotypes, reflecting less common glycosylation variations in the dataset (Figure 3). Amino acid alignment of representative ORF5 sequences showed variation at key epitope-associated, predicted N-glycosylation, and positively selected sites among the major PRRSV-2 lineages and variants. The detailed amino acid alignment is provided in Supplementary Figure S2.

### 3.5. Vaccine-like and Wild Strains Classification Based on ORF5 Similarity

Phylogenetic analysis of 642 ORF5 sequences, including vaccine-like and wild PRRSV-2 sequences, with strong bootstrap support (65–100), showed that 17.6% (113/642) were vaccine-like. Specifically, 82 sequences were classified as Ingelvac PRRS MLV (2-5-2) within the L5A.1 sub-lineage, with nucleotide and amino acid identities ranging from 97.52% to 99.83% relative to the reference vaccine. Amino acid comparison with the Ingelvac PRRS strain revealed only three nonsynonymous substitutions: one at residue 34 (A34D) in the HVR-1 (32–35 aa), a second at residue 141 (H141Y), and a third at residue 151, in the T cell epitope (149–163 aa). Additionally, the 82 sequences shared an identical N-glycosylation pattern—N30, N33, N44, and N51—matching the Ingelvac PRRS MLV strain’s ORF5 sequence.

Approximately 4% (3.89%; 25/642) of newly detected ORF5 sequences clustered with Prevacent PRRS (1-8-4), with nucleotide identity and amino acid identity ranging from 97.52% to 100%. All 25 ORF5 sequences were classified within the L1D.2 sub-lineage. Amino acid analysis showed no mutations in immunogenic regions of ORF5 compared with the Prevacent PRRS vaccine, except in 5 of 25 sequences (5/25). These five sequences exhibited nonsynonymous substitutions at residues 32, 33, and 35 in the HVR-1 region, plus an extra mutation at residue 17 (F17L). Moreover, three ORF5 sequences (0.46%; 3/642) were identified as PRRSGard-like (1-8-4) vaccine sequences with nucleotide identity ranging from 98.01% to 100%, belonging to the L1F sub-lineage. Another 2 ORF5 sequences (0.31%; 2/642) were classified as Fostera PRRS-like (1-3-2) vaccine sequences with nucleotide identity ranging from 98.84% to 99.17%, belonging to L8C.1. One sequence (0.15%; 1/642) was identified as PrimePac PRRS vaccine, with approximately 99.34% identity (Table 2). Conversely, 529 out of 642 (82.39%) ORF5 sequences were identified as wild-type or wild-like PRRSV-2 sequences. Most of these wild strains, from the 529 ORF5 sequences, are grouped into four distinct lineages: L1C, L1A, L1H, and L1E, consisting of 428, 53, 45, and 3 sequences, respectively.

## 4. Discussion

PRRSV is a serious pathogen that causes significant economic losses to the global swine industry by infecting both the respiratory tract and reproductive organs of pigs. The persistence and severity of the infection are based on several factors. Firstly, prolonged co-rearing of pigs, along with the presence of multiple age groups in the same environment, creates conditions conducive to PRRSV survival and continuous circulation. Given PRRSV’s ability to be transmitted vertically or horizontally, potential subclinical cases can shed the virus for extended periods, increasing the risk of recurrent infections and the coexistence of multiple strains within the same farm [35]. Nearly 30 to 50% of the American swine farms showed active circulation of PRRSV-2 with variable degrees of infection severity [36]. Secondly, PRRSV exhibits a high mutation rate [37], as do other RNA viruses. These genetic mutations primarily originate from high polymerase enzyme errors [38], lack of proofreading [39], and recombination events between various PRRSV-2 strains [17,39]. Therefore, all the previous conditions help the virus drive continuous evolution and genetic diversity, leading to extensive immune evasion, the cyclic emergence of most frequently detected new variants every few years, and failures in control and prevention strategies. In this study, we aimed to describe the ORF5 diversity of PRRSV-2 among diagnostic submissions from one of the largest pig-producing regions in the USA.

The phylogenetic analysis of ORF5 sequences collected from Midwest pig farms, typed by lineage and sub-lineage, showed aligned monophyletic clusters consistent with previous classifications [26,40]. It was specifically consistent with the recently proposed classification by Yim-im et al. [25], which divided PRRSV-2 into 11 lineages (two more than in previous classifications). The L1C sub-lineage was subdivided into five groups, plus one undefined group, whereas in previous classifications it was unsubdivided [25]. The phylogenetic analysis supported ORF5-based lineage assignment, although bootstrap support varied across nodes. The dataset showed that L1 was the most frequently detected lineage, comprising 86.76% (557/642) of sequences, which agreed with most previous studies, indicating that L1 has been the prevalent lineage among pig farms in the USA since 2005 [26,37,40]. Only 12.77% (82/642) of the total ORF5 sequences were assigned to the L5A lineage. This also agreed with several investigations conducted on PRRSV-2 in the USA, which reported that the detection of the L5 lineage in pig farms was sharply lower than that of the L1 lineage [25,41]. On the contrary, the dominant PRRSV-2 lineage in Europe is L5, with only a small number of samples classified as L1. This is supported by a study conducted in Hungary, which revealed that 88% of PRRSV-2 sequencescollected from pig farms between 2005 and 2020 were aligned with the L5 lineage, while only 12% were aligned with L1 [40].

Notably, in this study, the predominant sub-lineage in L1 was L1C, accounting for 66.66% (428/642) of the total ORF5 sequences. Nearly all these sequences clustered with L1C.5 (371/428), a novel 1-4-4 variant that emerged in 2020 and has shown a sharp increase among ORF5 sequences from pig farms in the United States. In contrast, only 8.25% (53/642) of the ORF5 sequences were identified as related to the L1A sub-lineage. This aligns with the finding that the L1A sub-lineage was predominant among diagnostic submissions in most US swine farms until 2020 but declined sharply by October 2020. Meanwhile, the number of pig farms reporting infection with the novel L1C.5 variant (1-4-4 RFLP pattern), especially in the Midwest, including Iowa and Minnesota, increased significantly [42]. Our findings are consistent with increased detection of L1C.5-related sequences among submissions from the Upper Midwest, including South Dakota; however, this diagnostic-submission dataset alone cannot establish the Upper Midwest as a population-level hotspot or determine herd-level infection burden. However, a recent study in Ohio and neighboring states contradicted our results, indicating that from 2017 to 2022, L1A followed by L5A were the most frequently detected sub-lineages among swine farms in these regions. This highlights the importance of considering regional factors when analyzing PRRSV-2 dynamics and evolution [41].

To refine the typing of the detected ORF5 sequences in this study, we used the online PRRSLoom-variants web tool [34], which further classified the detected sub-lineages and groups into variants. This indicated that seven variants have been designated for the L1C.5 group L1C.5.32 through L1C.5.38. We observed a shift among submitted sequences from classical L1C.5 in 2023 toward increased detection of L1C.5.32 in 2024 and 2025, especially in Minnesota, which was the most frequently detected variant in 2025 (*n* = 112), within the submitted dataset. Additionally, L1C.5.33, 34, and 36 have been detected among submitted sequences, totaling 19, 39, and 25, respectively, over the three years. The L1C.2 variant was detected more frequently among submitted PRRSV-2 sequences, especially in 2025. The L1C.3.25 variant was also detected in this study (*n* = 14). The L1H.18 variant, although first detected in 2018, has been reported less frequently in prior sequence datasets. We identified a notable increase in the number of detections of this variant, especially in 2025 (*n* = 17), compared with other variants in the L1H sub-lineage. Interestingly, the emergence of new variants in the L1C.5, L1C.2, and L1H have been reported as an increasing trend in variant circulation patterns in sequence-surveillance datasets, as recently illustrated by (https://Mshmp.Umn.Edu/PRRS-Variant-Monitoring) (accessed on 15 May 2026) [43].

In this study, the identity between the newly detected ORF5 and the reference strains was nearly consistent with the standard identity reported by Yim-im et al. (2023) [25], and the distance analysis within the created groups was also consistent. However, within sub-lineage L1H, we observed a marked increase in sequence divergence, with identity decreasing to around 88%, and the phylogenetic tree topology resolving three clearly separated branches within this sub-lineage. The deduced amino acid alignment revealed multiple amino acid substitutions among the aligned L1H strains, particularly in immunogenic sites, including T- and B-cell epitopes and decoy and neutralizing epitopes. Interestingly, each identified variant within the L1H sub-lineage contains specific mutations in these epitopes. These bioinformatic findings indicate molecular divergence within this sub-lineage and generate hypotheses about potential antigenic variation. It is important to validate these results through serological assays and animal experiments to assess the potential for antigenic change or reduced vaccine protection; ORF5 sequence data alone cannot demonstrate vaccine failure or immune evasion.

The analysis of variant distribution across years and locations revealed patterns of persistence, detection, and turnover, suggesting that PRRSV-2 ORF5 diversity among Midwestern diagnostic submissions was dynamic from 2023 to 2025. Sub-lineage L1C.5 was consistently detected across all years, suggesting repeated detection within the submitted dataset. The increase in L1C.5.32 detections, together with fewer classical L1C.5 detections, showed a temporal pattern within submitted sequences. Similarly, lineage L5A.1 grew over time (2023: 11.54% (24/208), 2024: 13.08% (17/130), 2025:13.49% (41/304)), which may reflect broader circulation, differential testing, or submission patterns. However, some variants, such as L1C.5.36, occurred only in 2025 (*n* = 25), possibly representing localized circulation, a newly detected variant, or changes in sampling or classification sensitivity. A limitation of this study is that the dataset was derived from diagnostic submissions and did not include the total number of submissions, the number of farms sampled, herd-level sampling intensity, or pig population denominators. Therefore, changes in annual detection counts should not be interpreted as true changes in PRRSV-2 incidence or infection rate. Instead, the observed patterns reflect changes in the composition of ORF5 sequences obtained from submitted RT-PCR–positive diagnostic samples. Moreover, the increase in submitted sequences in 2025, together with a shift in the frequently detected strains, can be examined from an epidemiological standpoint. This pattern may reflect increased incidence or transmission, greater diagnostic testing and sampling, changes in reporting, or multiple concurrent factors among submitted diagnostic samples. Still, the higher number of 2025 detections should be interpreted as increased detection in this dataset unless the denominator, herd-level, and repeat-sampling data are added. Thus, continuous monitoring of detected PRRSV variants in the U.S. swine industry is highly recommended, with an expanded geographical scope.

Geographic summaries at the sub-lineage and variant levels showed that most detections came from the same states responsible for overall detections, consistent with submission-based geographic concentration. The most frequently detected variants were centered in MN and SD submissions, indicating that genetic diversity and temporal variation were not uniformly distributed across the U.S. but were concentrated in submissions from the Upper Midwest. These findings have key epidemiological implications: first, they suggest concentration of detections in Midwest submissions, but disease burden and hotspot status cannot be confirmed without denominator, herd-level, and repeat-sampling data. Second, they indicate that the perceived “dominance” of certain sub-lineages might reflect actual circulation patterns combined with differences in surveillance effort, where repeated or intensive sampling in specific areas could skew the overall dataset.

The ORF5 classification based on vaccines and wild strains revealed that only around 20% of the ORF5 sequences detected among Midwest diagnostic submissions were vaccine-like strains: (12.77% Ingelvac PRRS MLV vaccine-like, 3.89% Prevacent PRRS-like strains, 0.46% for PRRSGard-like, 0.31% for Fostera PRRS-like) and 0.16% for Prime Pac PRRS-like. In contrast, the remaining 82.39% were classified as wild strains, including the L1C sublineage (predominantly L1C.5) and L1A, L1E, and L1H. Although commercially modified live vaccines used in American pig farms naturally produce strong immunity against homologous infections [44], they offer limited protection against heterologous infections [44,45]. Our study provides a descriptive summary of PRRSV-2 ORF5 diversity among Midwest diagnostic submissions and may help frame hypotheses about the increase in submitted detections, although this trend likely reflects multiple factors beyond vaccination, including differences in vaccine use and reliance on biosecurity alone. This is primarily due to the circulation of wild-type or wild-like PRRSV-2 sequences, particularly the novel L1C.5 variants, which are phylogenetically divergent from the modified live vaccines used [46]. However, ORF5 sequence similarity alone cannot determine vaccine mismatch, vaccine origin, or recombination between vaccine and field strains; such questions require vaccination-history data and whole-genome sequencing.

Regarding the lineage and sub-lineage classification, the amino acid alignment of ORF5 sequences within the same sub-lineages and groups, along with the reference sequences, revealed multiple nonsynonymous mutations in various immunogenic regions, including the signal peptide, the decoy epitope, B epitopes (1–15 aa and 187–200 aa), and HVR-1. Substitutions in these immunogenic regions in the GP5 have been associated with altered antigenic properties in prior studies [11,47]. The unique substitutions at positive selection sites (102, 104, and 151 aa) and in the terminal B epitope (187–200 aa), observed in the newly detected ORF5 sequences and in the reference genomes, could primarily be associated with antigenic shift and variation [13,15].

Various N-glycosylation patterns were identified across the 642 ORF5 sequences analyzed in this study, totaling 30, with five accounting for approximately 79% of the detected strains. Only N44 and N51 were conserved throughout the sequences, whereas significant variation was observed in ORF5 sequences at other sites, including N30, N32, N33, N34, N35, and N57, with two additional novel sites detected at positions 59 and 191. These variations in N-glycosylation patterns among ORF5 sequences may contribute to structural and functional modifications of the viral envelope glycoprotein 5 (GP5). Previous studies have demonstrated that the addition or removal of N-glycosylation sites is associated with changes in PRRSV-2 antigenicity and pathogenicity [47]. Alterations in these glycosylation sites can also influence GP5 conformation, facilitating evasion of the host immune response and reducing the effectiveness of neutralizing antibodies [16,17]. Overall, aligned sequences within the same sub-lineages and groups showed characteristic substitutions in immunologically relevant regions of GP5, including epitope-associated sites. The subsequent variant and subgroup classification further indicated that several variants carried distinct amino acid changes in these regions, suggesting potential antigenic variation even among viruses assigned to the same broader lineage or sub-lineage group. In addition, the observed variation in glycosylation-associated motifs, epitope-related sites, and codons under positive selection supports the biological relevance of ORF5-based surveillance beyond routine lineage assignment. Because GP5 is one of the major immunogenic proteins of PRRSV-2, changes in this region may influence antigenic diversity, immune recognition, and the persistence of specific viral variants in the field. In the present study, these molecular features were interpreted together with lineage, yearly, and geographic patterns to provide a broader understanding of PRRSV-2 diversity among the analyzed sequences. At the same time, these findings highlight an important limitation of relying exclusively on ORF5-based Sanger sequencing for PRRSV-2 characterization. Although ORF5 remains highly useful for routine lineage classification, vaccine-relatedness assessment, and large-scale diagnostic surveillance, it represents only a small portion of the PRRSV genome and may not fully capture recombination events or discordant evolutionary histories occurring in other genomic regions.

A recent whole-genome sequencing study of PRRSV-2 isolates showed that several viruses had different phylogenetic placements when analyzed by ORF5 alone versus whole-genome sequences, with the discordance explained by recombination events involving regions outside ORF5 [48]. This highlights an important limitation of the present study, which was based solely on ORF5 sequences rather than whole-genome data. Although ORF5 remains a valuable target for routine PRRSV molecular epidemiology, lineage classification, and surveillance, analysis of a single genomic region provides limited resolution for identifying recombination breakpoints, determining parental strains, or characterizing genome-wide mosaic patterns. Therefore, any recombination-related observations in this study should be considered preliminary and interpreted cautiously, and recombination was not treated as a primary finding. Similarly, the amino acid substitutions and glycosylation patterns observed in the ORF5 dataset should be viewed as useful indicators of potential antigenic variation, but not as a complete representation of viral evolution. Future studies incorporating whole-genome sequencing will be essential to validate potential recombination events, evaluate variation in non-ORF5 genomic regions, reduce potential bias associated with targeted amplicon sequencing, including primer-binding-site mismatches in divergent strains, and provide a more comprehensive understanding of the evolutionary dynamics of circulating PRRSV-2 strains.

## 5. Conclusions

In conclusion, this study applies the new, refined classification of PRRSV-2 proposed by PRRSLoom to ORF5 sequences collected from the clinical samples submitted from Midwest swine farms to the SDSU-ADRDL during 2023–2025. However, it did not represent nationwide distribution. First, this study found that L1C.5-related variants were the most frequently detected sequences in this Midwestern diagnostic-submission dataset; transmissibility and virulence cannot be inferred from ORF5 surveillance alone. This study highlighted a notable shift in submitted PRRSV ORF5 sequences, with the emergence of new variants, including L1C.5.32, L1C.5.34, L1C.5.36, L1C.3.25, L1C.2, and L1H.18, particularly in 2025 submissions. L1H showed marked variability and divergence, even at the amino acid level, requiring further, more refined analysis. Notably, more than 82.39% of the detected ORF5 sequences in Midwest swine farms were wild-like strains with low identity to commercial vaccines, with L1C.5-related sequences representing the largest group. This may help generate hypotheses about the increase in submitted detections in 2025, underscoring the importance of molecular characterization of field strains to track viral evolution and improve disease control and vaccine development. High variation was detected in the pattern of N-glycosylation sites across the ORF5 sequence, with only N44 and N51 highly conserved.

Because this study was based on diagnostic submissions, the reported lineage and variant frequencies reflect detection patterns among submitted RT-PCR–positive samples rather than true population-level prevalence, incidence, or infection rates. Additionally, this study identified an important point that has been overlooked in all previous classifications: amino acid changes in the immunogenic regions of ORF5. Despite high identity of ORF5 sequences within the same cluster with reference sequences and among themselves, multiple nonsynonymous substitutions have been observed in the B and T epitopes, HVRs, and glycosylation sites. This suggests possible antigenic diversity and variation among members within the same sub-lineage and group. Furthermore, this study underscores the importance of whole-genome sequencing for a broader understanding of PRRSV’s molecular evolution, including the characterization of additional viral proteins and a better understanding of viral evolution, antigenic change, and potential phenotypes. Whole genome sequencing can also address the bias in Sanger sequencing, which might miss highly variable strains due to primer mismatches when using low Ct samples.

## Figures and Tables

**Table 1:** The created sub-lineages and groups of the ORF5 sequences collected from Midwest swine farms and their identity percentages with reference sequences and average divergence.

Average Genetic Divergence Within Lineage			
Sub-Lineage	Number of Sequences (*n*)	Identity Range of NT (%)	Identity Range of AA (%)
L1C.5	371	91.87–100.0	90.5–100.0
L5A	82	96.68–100.0	94.0–100.0
L1A	53	90.55–100.0	89.5–100.0
L1H	45	85.74–99.83	88.0–100.0
L1C.2	35	83.08–100.0	82.5–100.0
L1D	25	97.01–100.0	95.0–100.0
L1C.3	17	91.21–99.83	91.0–100.0
L1A-unclassified	8	89.39–99.5	91.5–99.0
L1C-unclassified	5	89.05–96.02	89.5–95.5
L1E	3	88.89–98.51	89.0–97.0
L1F	3	97.84–99.83	96.0–100.0
L8	2	99.34–99.34	98.0–98.0
L7	1	-	-

**Table 2:** The percentage of vaccine-like and wild-like strains of the collected ORF5 sequences from Midwest pig farms.

	Percentage	Identity to Reference ORF5 (%)	Sublineage	RFLP Pattern
Ingelvac PRRS MLV-like	12.77% (82/642)	97.52–99.83	L5A	2-5-2
Prevacent PRRS-like	3.89% (25/642)	97.52–100	L1D	1-8-4
PRRSGard-like	0.46% (3/642)	98.01–100	L1F	1-8-4
Fostera PRRS-like	0.31% (2/642)	98.84–99.17	L8C	1-3-2
Prime Pac PRRS-like	0.16% (1/642)	99.34	L7	1-4-4
Wild-like strains	82.39% (529/642)			

## Data Availability

The ORF5 sequence data and associated lineage, variant, temporal, and geographic summaries generated and analyzed in this study are provided in Supplementary File S1. Additional information is available from the corresponding author upon reasonable request.

## Supplementary Materials

The following supporting information can be downloaded at: https://www.mdpi.com/article/10.3390/pathogens15070710/s1, Supplementary File S1: ORF5 nucleotide and amino acid sequences, reference/vaccine sequences, PRRSLoom lineage and variant assignments, and lineage-specific temporal and geographic summary tables for PRRSV-2 sequences analyzed in this study; Supplementary Figure S1: Collapsed ORF5 maximum-likelihood tree summarizing variant/sub-lineage assignments, sequence counts, and bootstrap support for frequent assignments; Supplementary Figure S2: GP5 amino acid alignment highlighting variable residues, predicted N-linked glycosylation sites, and key epitope-associated regions.
