# Supplementary material for: Geographic Distribution and ORF5 Diversity of PRRSV-2 Variants in Midwestern U.S. Diagnostic Submissions, 2023–2025
Source: Pathogens. 2026 Jul 7;15(7):710. doi: 10.3390/pathogens15070710 (PMC13414845; doi:10.3390/pathogens15070710)
Supplement: Supplementary file 1 [file pathogens-15-00710-s001.zip › Supplementary Figure S1.pdf]

A

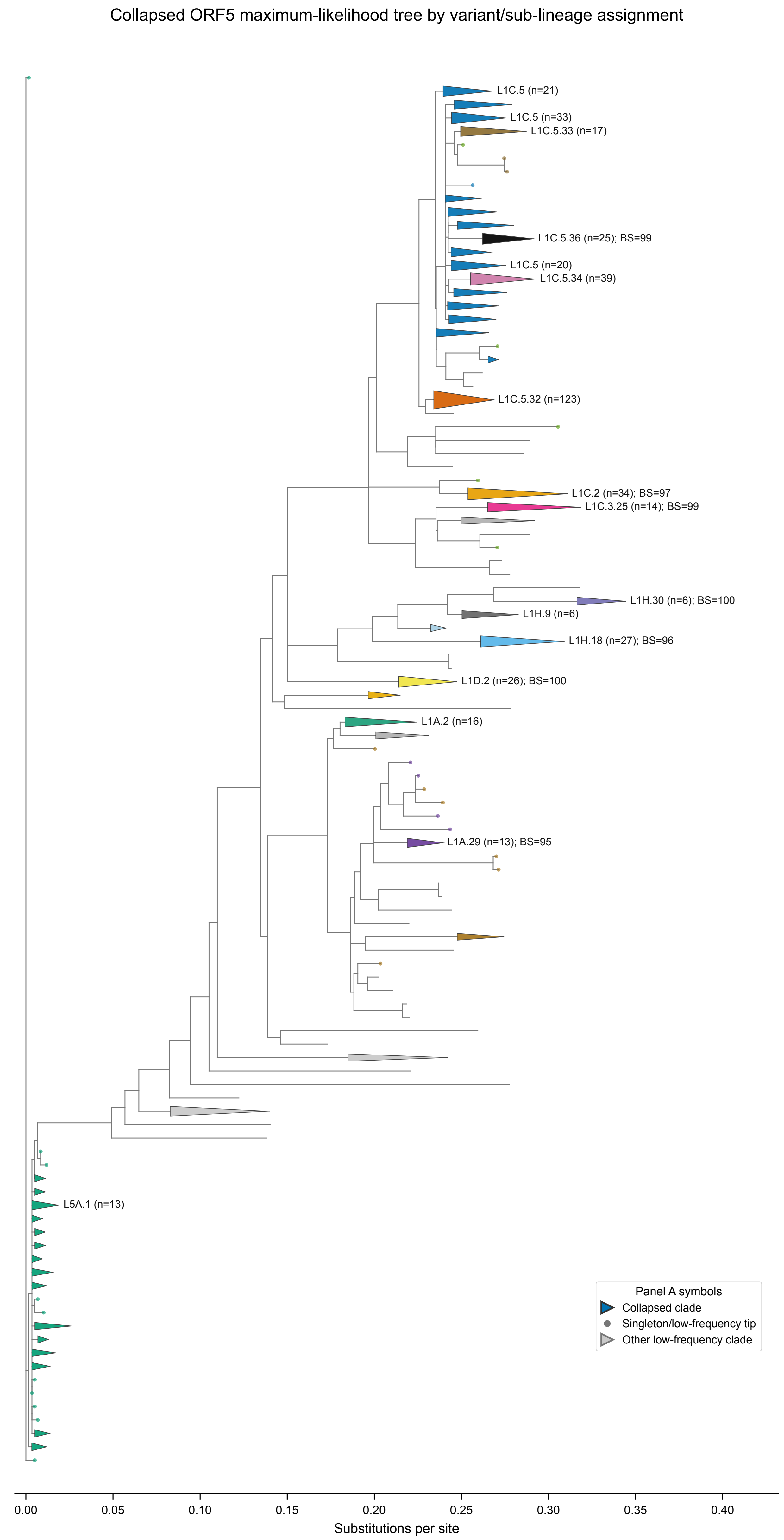

B

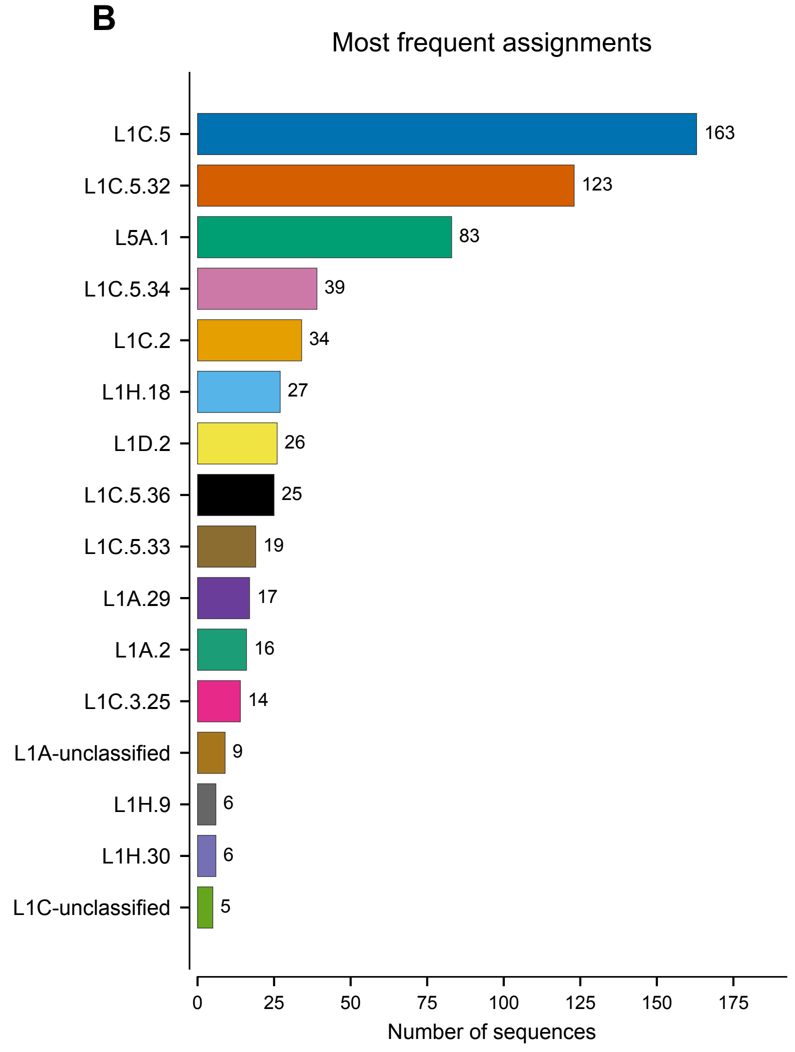

C

Support summary for frequent assignments

Assignment	n	Pure MRCA	MRCA BS	Separate pure clades (n>=3)
L1C.5	163	no	17	13
L1C.5.32	123	yes	47	1
L5A.1	83	no		15
L1C.5.34	39	yes	76	1
L1C.2	34	yes	97	1
L1H.18	27	yes	96	1
L1D.2	26	yes	100	1
L1C.5.36	25	yes	99	1
L1C.5.33	19	no	43	1
L1A.29	17	no	34	1
L1A.2	16	yes	27	1
L1C.3.25	14	yes	99	1
L1A-unclassified	9	no	45	1
L1H.9	6	yes	48	1
L1H.30	6	yes	100	1
L1C-unclassified	5	no	59	0

MRCA = most recent common ancestor; BS = bootstrap support.
Pure MRCA = all tips below the MRCA have the same assignment.
MRCA BS = ultrafast bootstrap support at that assignment's MRCA when available.
Groups with multiple separate pure clades, such as L1C.5 and L5A.1, are shown as repeated collapsed triangles in panel A rather than as one forced clade.
