# Supplementary material for: Geographic Distribution and ORF5 Diversity of PRRSV-2 Variants in Midwestern U.S. Diagnostic Submissions, 2023–2025
Source: Pathogens. 2026 Jul 7;15(7):710. doi: 10.3390/pathogens15070710 (PMC13414845; doi:10.3390/pathogens15070710)
Supplement: Supplementary file 1 [file pathogens-15-00710-s001.zip › Supplementary Figure S2.pdf]

| Position | 2 | 3 | 5 | 6 | 10 | 11 | 15 | 17 | 23 | 27 | 28 | 30 | 32 | 33 | 34 | 35 | 37 | 39 | 44 | 45 | 51 | 57 | 58 | 59 | 60 | 72 | 77 | 87 | 98 | 102 | 104 | 137 | 141 | 151 | 153 | 158 | 163 | 187 | 191 | 192 | 196 | 200 |
|---|---|---|---|---|---|---|---|---|---|---|---|---|---|---|---|---|---|---|---|---|---|---|---|---|---|---|---|---|---|---|---|---|---|---|---|---|---|---|---|---|---|---|
| **Site** | * | | * | * | | | * | | | | * | N* | N* | N* | N* | N* | | * | N | | N | N | | N* | | | | | | * | * | * | | * | * | | | | N | | * | |
| L1C.5 (n=162) | L | G | C | L | C | C | L | L | F | A | L | N | N | N | S | S | S | L | N | L | N | N | K | K | F | V | V | F | T | Y | G | S | Y | K | Y | S | K | T | K | I | Q | P |
| L1C.5.32 (n=123) | L | G | C | L | C | C | L | L | F | A | L | N | N | N | D | S | S | L | N | L | N | N | D | K | F | V | V | F | T | Y | G | S | Y | K | Y | S | K | T | K | I | Q | P |
| L5A.1 (n=82) | L | G | C | L | C | C | L | L | F | V | L | N | S | N | D | S | S | L | N | L | N | A | N | K | F | V | V | F | T | V | G | A | Y | R | Y | P | K | T | R | V | Q | P |
| L1C.5.34 (n=39) | L | G | C | L | C | C | L | L | F | A | L | N | D | N | S | S | S | L | N | L | N | D | K | R | F | V | V | F | T | Y | G | S | Y | K | Y | S | K | T | K | I | Q | P |
| L1C.2 (n=33) | L | G | C | L | C | C | L | L | S | A | L | N | N | N | S | S | S | L | N | L | N | N | N | K | F | A | V | F | T | Y | G | S | Y | K | Y | S | K | T | R | I | Q | P |
| L1H.18 (n=27) | L | G | C | L | C | C | P | L | F | A | L | N | D | N | N | S | S | L | N | L | N | N | N | H | F | A | V | F | T | Y | K | S | Y | K | Y | P | K | T | K | V | Q | P |
| L1D.2 (n=25) | L | G | C | L | Y | C | L | F | F | A | L | N | A | S | N | S | S | L | N | L | N | N | Q | K | F | V | V | F | T | Y | G | S | Y | N | Y | P | R | T | K | I | Q | P |
| L1C.5.36 (n=25) | L | G | C | L | C | C | L | L | F | A | P | N | N | N | S | S | S | L | N | L | N | N | K | K | F | V | I | F | T | Y | G | S | Y | K | Y | S | R | T | K | I | Q | P |
| L1C.5.33 (n=19) | L | G | C | L | C | C | L | L | S | A | L | N | N | N | S | S | S | L | N | L | N | N | D | K | F | V | V | F | I | Y | G | S | Y | K | Y | S | K | T | K | I | Q | P |
| L1A.29 (n=17) | L | G | C | L | C | C | H | L | F | A | I | N | N | N | S | S | S | L | N | L | N | N | K | T | F | V | V | F | A | Y | G | S | Y | K | Y | S | K | T | K | V | Q | P |

Orange cells differ from the L1C.5 consensus. N = predicted N-glycosylation motif start; * = positive-selection site discussed in the manuscript.

B cell epitope    Decoy epitope    HVR-1/HVR-2    Neutralizing epitope    T cell epitope    Other discussed site
